# Fall risk screening: Audiologists’ perceived knowledge, views and reported practice

**DOI:** 10.4102/safp.v67i1.6072

**Published:** 2025-04-23

**Authors:** Kayla J. McFarlane, Amisha Kanji, Alida Naude

**Affiliations:** 1 Department of Audiology, Faculty of Humanities, University of the Witwatersrand, Johannesburg, South Africa; 2 Centre for Augmentative and Alternative Communication, Faculty of Humanities, University of Pretoria, Pretoria, South Africa; 3 Division of Speech-Language and Hearing Therapy, Department of Health and Rehabilitation Science, Faculty of Medicine and Health Sciences, Stellenbosch University, Cape Town, South Africa

**Keywords:** audiology, fall-risk screening, older adults, perceived knowledge, clinical practice

## Abstract

**Background:**

Falls among older adults are a major public health issue. In South Africa, where the elderly population is expected to quadruple within the next three decades, fall prevention is critical.

**Methods:**

This study evaluated South African audiologists’ perceived knowledge, views and reported practices regarding fall risk screening (FRS) in older adults. A quantitative online survey was conducted using an adapted questionnaire designed to assess various aspects of FRS practice. Data were analysed using descriptive and inferential statistics.

**Results:**

The survey received responses from 106 audiologists. Most respondents reported using familiar tools, such as case history and vestibular assessments, to conduct FRS, with fewer utilising functional balance measures. Only 11% of audiologists reported prior knowledge of FRS, while 69% – 74% perceived their knowledge as insufficient to screen or counsel older adults. While 58% recognised FRS as part of the audiologist’s scope, only 21% felt comfortable conducting it. Key barriers included insufficient training (80%) and time constraints (48%). Despite this, 98% expressed interest in learning about FRS and 90% believed FRS could enhance the profession.

**Conclusion:**

The findings underscore the need for improved clinical guidelines, educational initiatives and practice standards to better equip audiologists in fall prevention efforts and a collaborative approach to fall risk management for older adults.

**Contribution:**

This study emphasises the importance of incorporating FRS into undergraduate audiology curricula and continuous professional development programmes and encourages the use of a biopsychosocial approach and collaboration among multidisciplinary teams in fall risk management for older adults.

## Introduction

Falls among older adults are a major public health issue, with approximately 37 million falls reported annually, severe enough to require medical attention.^1^ By 2050, as older adults make up 15% of the global population, falls could escalate to an estimated 385.6 million incidents.^2^ Older adults include all adults over the age of 65 years. This is in line with the American Geriatrics Society (AGS) and British Geriatrics Society (BGS) guideline which recommends annual fall risk screening (FRS) for all adults aged 65 years and older.^3^

The World Health Organization (WHO) defines a fall as: ‘an event whereby an individual unexpectedly comes to rest on the ground or another lower level’(p. 3)^4^, ‘which is not the result of a major intrinsic event or external hazard (e.g. myocardial infarction, stroke, seizure or an overwhelming external hazard, such as being hit by a vehicle)’(p. 3)^5^. A person is said to have a fall risk when one or multiple fall risk factors are evident.^6^

Older adults seeking audiological care are at higher risk of falls because of age-related inner ear sensory deterioration.^7,8^ Research has indicated older adults with unaddressed mild hearing loss exhibited an almost threefold increase in the likelihood of having a history of falls. Furthermore, for every 10 decibels that a patient’s hearing deteriorated, their chances of falling increased by 1.4 times.^9^ Criter and Honaker^10^ found that 50% of older adults seen in an audiology hearing clinic reported at least one fall within a year. The consequences of falls are not only immediate but also encompass a wide array of medical, physical, psychological, social and financial repercussions, underscoring the urgent need for effective fall prevention strategies.^11^

Conversely, protective factors can mitigate fall risk. These factors include maintaining physical activity to strengthen muscles and improve balance,^12^ engaging in balance-specific exercises such as Tai Chi^13^ and correct use of assistive devices.^14^ Modifications to the home environment, including installing grab bars and ensuring adequate lighting, are proven strategies for fall prevention.^15^ In addition, regular health checkups to address vision and hearing impairments^16^ and optimise medication use^17^ can significantly reduce fall risk. Incorporating these considerations into clinical guidelines and educational initiatives can equip audiologists and other healthcare providers with the tools to adopt a holistic approach to fall prevention, addressing both risk and protective factors.

In South Africa (SA), where the elderly population is expected to quadruple within the next three decades, fall prevention is critical.^18^ Despite their expertise, audiologists underutilise FRS, with less than 17% feeling adequately trained.^19^ This gap is even more pronounced in sub-Saharan Africa, where literature on FRS by audiologists is scarce,^20^ and no studies have specifically examined the knowledge, views and practices of South African audiologists in this domain. The ethical implications of this gap are profound. If FRS is within the audiologist’s scope of practice and not being conducted, it raises concerns about the principles of beneficence and non-maleficence. Hence, the need to explore its integration into clinical practice.

In addition, the previous outdated Health Professions Council of South Africa (HPCSA) guidelines exacerbate this issue. The HPCSA guidelines have not incorporated recent global advances in fall prevention strategies nor adequately address the expanded role audiologists can play in multidisciplinary fall risk management. Studies such as those by Montero-Odasso et al.^21^ and Greene et al.^22^ have emphasised the importance of updated guidelines to include FRS as a routine part of audiological care. In their current form, the HPCSA guidelines limit the scope of FRS, missing opportunities for early intervention and cross-disciplinary collaboration. The HPCSA has only recently (in 2024) stated that fall risk has been included within the regulations relating to the scope of practice for audiologists, although no official documentation is available on the professional board’s website at present (Mazinyo, T., 24 October 2024, letter, Committee Coordinator for the Professional Board for Speech Language and Hearing Professions).

Fall risk screening does not require extensive training and can be efficiently integrated into clinical practice with tools such as the Tinetti Assessment Tool, which are low-cost and easy to administer, requiring only 10–15 min to administer.^23^ Given that the success of fall prevention is closely tied to the clinician’s knowledge of fall risk factors,^24,25^ it was critical to assess the current state of knowledge among South African audiologists and their views on incorporating FRS into their practice. This study aimed to evaluate the perceived knowledge, views and reported practices of South African audiologists regarding FRS in older adults. In addition, the study aimed to identify barriers to the implementation of FRS and highlight opportunities for integrating FRS training into clinical practice and educational curricula. These objectives frame the analysis and interpretation of the results, guiding recommendations for future action.

## Method

### Study design

A cross-sectional, quantitative survey design was used.

### Participant recruitment and study sample

Participants comprised of audiologists registered with the HPCSA who had experience with adult patients. A convenience sampling approach was used to recruit registered audiologists working with older adults in South Africa. Participants had to be registered with the HPCSA, and have adult patients in their caseload or have had experience with adult patients. Recruitment was conducted through professional networks, online platforms and emails sent via audiology professional associations and social media channels. These networks or platforms included the South African Association of Audiologists (SAAA), the National Speech Therapy and Audiology Forum and relevant social media platforms such as Facebook, LinkedIn and WhatsApp. These channels were utilised to disseminate an infographic that comprised of information about the study, invited participation and provided the link to the survey. This approach ensured outreach to a diverse pool of audiologists actively involved in clinical practice.

### Sample size calculation

The sample size was calculated based on the total number of audiologists registered with the HPCSA as of 2021. At the time, there were 1485 registered audiologists, including dually qualified speech therapists and audiologists. Using an estimated population size of 1485, a confidence level of 95% and a margin of error of 5%, a minimum sample size of 306 audiologists was determined.

### Data collection

An online survey was administered using Google Forms, with the survey link being open from 01 March to 30 May 2022.

### Survey instrument

The survey was adapted from Patterson and Honaker’s^19^ study to align with South African audiology practice, informed by a systematic review.^26^ Data from the systematic review were compared to the original survey, and additional ideas and/or concepts pertaining to audiologists’ perceived knowledge, views and reported practice were added to the survey. The survey was adapted to include the most regularly reported tools as options for audiologists to select when reporting which tools they regularly included within FRS protocols. Some questions were removed or added to ensure that they align to the research question and rephrased where necessary to avoid ambiguity. Contextual changes were also made, such as changes to terminology. A pilot study of the adapted survey was then conducted with six audiologists, varying in work sector (public and private), English as their first language and FRS experience, and provided feedback on the survey’s structure, content and usability, which was used to further refine the survey. Overall, three changes to the questions were made based on participant suggestions. Namely, three questions were adapted to reduce bias within the question; six questions were adapted to highlight subtle differences within the questions; and three questions were reworded to ensure that participants were answering based on their personal views and not on a generalised view of audiologists.

The survey consisted of 50 questions. Out of these 50 questions, 27 were multiple-choice, 19 were scored on a 5-point Likert-scale (strongly agree, agree, uncertain, disagree, strongly disagree) assessing audiologists’ views, perceived knowledge and reported practices related to FRS, and four were open-ended questions related to the number of years of experience, hours of FRS education and the approximate time thought to be taken to conduct FRS.

The use of an online survey had several advantages. These methods enabled the collection of data from a geographically diverse sample, which was particularly important given the representation across all nine provinces in South Africa.^27^ Online distribution also facilitated accessibility and reduced logistical challenges associated with in-person data collection.^28^ In addition, the structured nature of the survey allowed for consistent data collection, enhancing comparability across respondents.^29^ However, there were also notable disadvantages. The reliance on self-reported data may have introduced response bias, as participants could overestimate or underestimate their knowledge and practices.^30^ The online platform may have excluded potential participants who lack internet access or are less familiar with digital tools, thereby introducing selection bias.^31^ Furthermore, the lack of real-time interaction limited the ability to clarify ambiguous responses or probe deeper into participants’ perspectives.

### Data analysis

Data were analysed quantitatively using descriptive and inferential statistics. Descriptive statistics were used to summarise participant responses. Inferential statistics were employed to explore relationships between variables. All statistical analyses were conducted using RStudio, and a significance level of 0.05 was applied to all tests.

### Reliability and validity

To ensure construct validity, each section of the survey was linked to the specific objectives of the study. A systematic review of the literature exploring fall risk assessment in audiology was conducted. The survey was further adapted based on these findings to ensure that it included pertinent points on FRS which were identified in audiology literature. This strengthened the content validity of the research tools. A pilot study was conducted to ensure content and face validity, and to determine whether the methodology, sampling instrument and analysis were adequate. Research data and findings were reviewed by the two co-authors with knowledge in fall risk and research. This improved the credibility of the data analysis and interpretations.^32^

### Ethical considerations

The study received ethical approval from the University of the Witwatersrand’s Human Research Ethics Committee (Non-Medical) (Protocol No. H20/09/56). Participation in the survey was voluntary, and written informed consent was obtained electronically from all participants before they began the survey. Participants were assured of the confidentiality and anonymity of their responses, and no identifying information was collected.

## Results

### Demographics

A total of 106 audiologists across SA participated in the online survey. In SA, 1485 audiologists are reportedly registered with the HPCSA (including dually registered speech therapists and audiologists)^33^. This dataset represents approximately 7% of the audiologist population in SA. Using Cochran’s formula with a confidence level of 95%, the margin of error was calculated at 9.2%.

Demographic information is summarised in Table 1 for ease of reference.

**TABLE 1:** Demographic information of survey participants.

Category	Sub-category	*n*	%
Total participants	-	106	-
Registered audiologists	-	1485	7.0
Qualifications	STA	-	50.0
	AU	-	50.0
Time since qualification	Qualified in the last decade	-	62.0
Highest qualification	Undergraduate degree	-	77.0
University	SMU	-	8.5
	University of Cape Town	-	16.0
	University of KwaZulu-Natal	-	8.5
	University of Pretoria	-	42.4
	University of the Witwatersrand	-	24.5
Sector	Private healthcare	-	55.0
	Public healthcare	-	41.0
Regional representation	All nine provinces	-	100.0
Services provided to older adults	Diagnostic audiological assessments	-	93.0
	Hearing aid fittings	-	93.0
	Balance or vestibular services	-	37.0

AU, Audiology; HPCSA, Health Professions Council of South Africa; SMU, Sefako Makgatho Health Sciences University; STA, Speech Therapy and Audiology.

The most commonly provided services to older adults included diagnostic audiological assessments (93%) and hearing aid fittings (93%). Balance or vestibular services (screening, diagnostics and rehabilitation) were less commonly provided (37%).

### Perceived knowledge of fall risk screening

Only 11% of the participants indicated prior knowledge on FRS, with 50% indicating that they had a limited amount and 39% indicating no prior knowledge. Between 69% and 74% of the participants perceived their knowledge as insufficient to identify, screen or counsel older adults at risk of falling. Of participants with insufficient knowledge, 27% were providing vestibular services at the time of the survey.

Participants were asked about their knowledge of the International Classification of Functioning, Disability, and Health (ICF)^34^ as a guideline for FRS. Only 21% perceived their knowledge of the ICF as sufficient. Participants who reported sufficient knowledge of the ICF were more likely to conduct FRS, with a statistically significant *p*-value of 0.009 and a Chi-squared statistic of 11.51.

**FIGURE 1:**
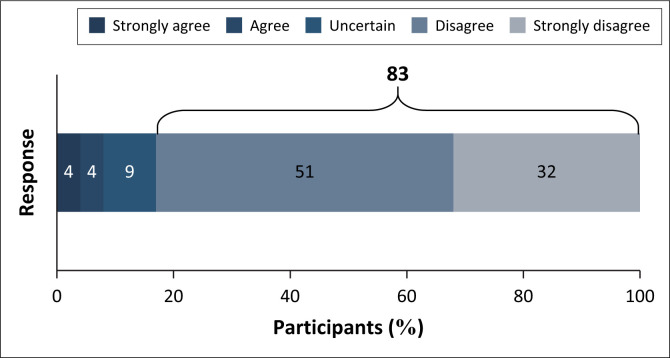
Audiologists are sufficiently trained to conduct fall risk screenings in undergraduate curricula (*N* = 106).

Less than 8% of the participants agreed that audiologists receive sufficient training in FRS during undergraduate (UG) studies (Figure 1).

**FIGURE 2:**
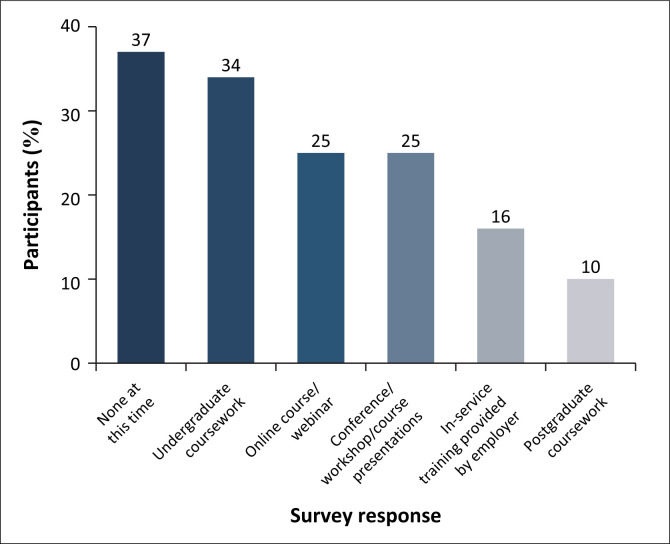
Professional preparation that focused on fall risk screening (*N* = 106).

While 19% of the participants reported having the opportunity to learn about FRS during their undergraduate training, only one-third of those considered the training to be adequate. Further, 32% reported opportunities to learn about FRS after graduation, with the most common postgraduate learning opportunities being online courses/webinars (25%) and conferences/workshops/courses (25%) (Figure 2).

**FIGURE 3:**
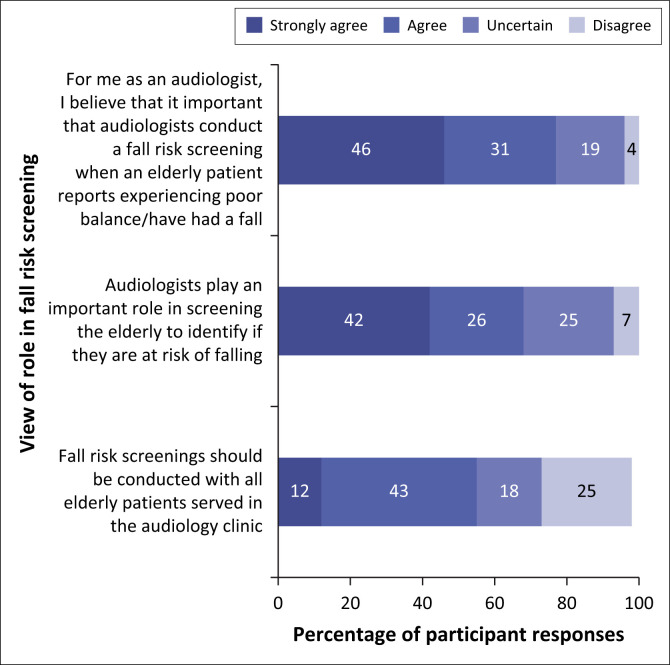
Survey participants’ views on the audiologist’s role in fall risk screening (*N* = 106).

In all, 58% of the participants believed that FRS is within the audiologist’s scope of practice, while 10% perceived it as outside the scope. Further, 77% agreed that consulting audiologists should conduct FRS if an older adult reports imbalance, and 68% agreed that audiologists play an important role in FRS (Figure 3). However, only 21% felt comfortable identifying someone as ‘at-risk of falling’.

**FIGURE 4:**
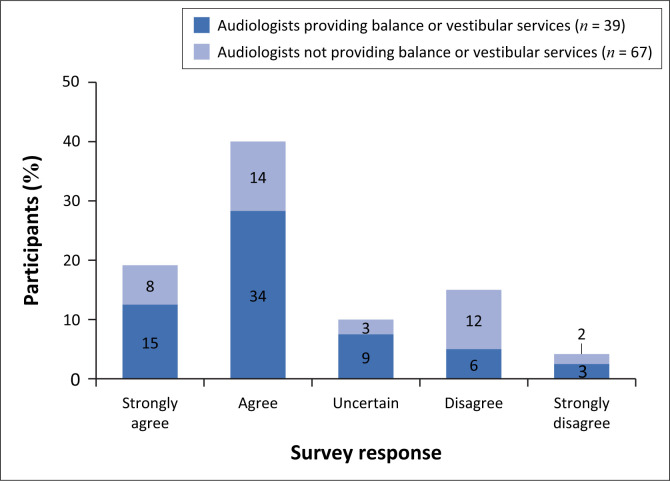
Fall risk screening services should be more prevalent in the vestibular or balance disorder clinical setting compared to the ‘general’ audiology clinical setting (*N* = 106).

Participants were asked about the appropriate clinical setting for FRS. While 67% indicated that FRS should primarily be conducted in vestibular settings, 71% believed it should also be conducted in general hearing clinics (Figure 4). In all, 98% of the participants expressed interest in further training on supporting older adults at risk of falling, and 96% found learning new FRS methods helpful. Majority of the participants (92%) believed that FRS is important for addressing the needs of the ageing population.

Almost all (98%) participants expressed interest in learning more about how to support older adults who are at risk of falling and 96% agreed that learning new ways to administer FRS would be helpful to them. Further, 92% believed FRSs were important in addressing the needs of our ageing population and 83% perceived that all older adults could benefit from FRS. The expressed benefits of FRS included decreasing the number of individuals who may experience a falling event (90%) and minimising any associated negative consequences a fall may have on a patient’s life (82%). The perceived importance of FRS was further highlighted with 95% of the participants agreeing that it may have a significant impact on their patients’ future quality of life.

**FIGURE 5:**
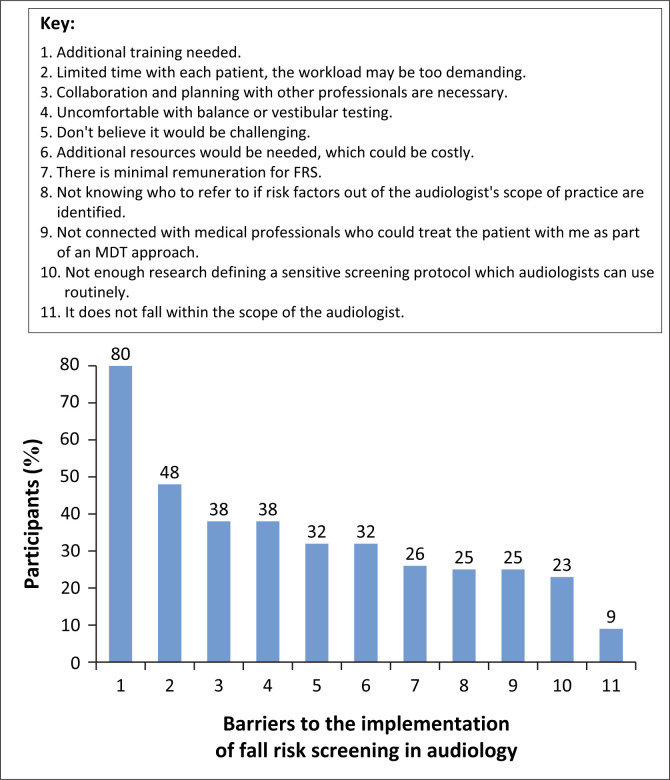
Fall risk screening implementation barriers for audiologists (*N* = 106).

Majority of the participants (89%) agreed that if audiologists had knowledge of FRS, it would change how ‘at-risk’ patients are identified and assessed in both the hearing and vestibular clinic environments. These changes appeared to be viewed positively, as 90% of the participants also believed that FRS implementation may offer opportunity for expansion of the audiology profession. Despite this positive view, several perceived barriers to the implementation of FRS in clinical practice were reported (Figure 5).

The most commonly perceived barrier reported was audiologists’ need for additional training (80%). This correlated with an earlier finding whereby participants reported limited training on FRS. Concerns regarding time constraints and workload were the next most reported barrier (48%). Participants also indicated that collaboration and planning with other professionals could be challenging (38%). Participants associated FRS with vestibular practice competency, where 38% of the participants indicated that they were not comfortable with balance or vestibular testing and would therefore not feel comfortable with FRS (38%). Several participants (17/67 [25%] non-vestibular and 17/39 [44%] vestibular audiologists) believed that FRS would not be challenging to implement.

### Reported practice of fall risk screening

Participants reported their actions when encountering older, adult patients with unsteady gait. While 45% referred patients to a physiotherapist, 55% did not take action, assuming the issue had been addressed by the patient’s General Practitioner (GP). Only 4% indicated conducting FRS in such cases.

Of those providing vestibular services, 11% reported conducting FRS often or always. Participants who conducted FRS most commonly used case history (82.5%), followed by observations of gait, steadiness and questions about fall history, fear of falling and dizziness or imbalance.

Just under half of the participants (49%) indicated that they had heard of FRS but were still not confident to implement it without further training. An additional 24% specified that they had never heard of or thought about conducting FRS as an audiologist. The Kruskal-Wallis test revealed that those who had never heard of FRS as part of the audiologists’ role were less likely to view FRS as being within the audiologist’s scope of practice, with a statistical significance of *p* = 0.000.

In all, 59% of the participants indicated that they never or rarely ask about history of falls and 83% indicated that they never or rarely ask about fear of falling in their case history session with older, adult patients.

Participants who stated that they always, often or rarely conduct FRS were given the opportunity to disclose which tools they would use when choosing to conduct an FRS (*n* = 40). The most commonly selected tools can be viewed in Table 2.

**TABLE 2:** Tools participants stated they would use when conducting a fall risk screening with elderly adults (*N* = 40).

Fall risk screening tools	*n*	%
1. Case history	33	82.5
2. Observation of patient’s gait and steadiness	22	55.0
History of falls or fear of falling	22	-
Presence of dizziness or imbalance	22	-
3. DHI	16	40.0
4. Fukuda stepping test	13	33.0
BPPV assessment	13	-
5. Family member interviews about falls	11	28.0
Gait assessment	11	-
6. mCTSIB	10	25.0
DVA	10	-
7. VNG/ENG	9	22.0
8. TUG/DGI	8	20.0
HHIE	8	-
VEMPS	8	-
GST	8	-
9. Questions about inactivity	7	18.0
Caloric	7	-
10. Use of ambulatory device	5	13.0
Thirty second sit-to-stand	5	-
vHIT	5	-
Vision screening	5	-

Note: All other fall risk screening tool options were selected by less than 12.5% of the participants. Percentages do not add up to 100% because participants selected multiple tools.

BPPV, Benign Paroxysmal Positional Vertigo; DGI, Dynamic Gait Index; DHI, Dizziness Handicap inventory; DVA, Dynamic Visual Acuity; GST, Gaze Stabilisation Testing; HHIE, Hearing Handicap Inventory for the Elderly; mCTSIB, Modified Clinical test of Sensory Integration on Balance; TUG, Timed Up and Go; VEMPS, Vestibular Evoked Myogenic Potentials; VNG/ENG, Videonystagmography/Electronystagmography; vHIT, Video Head Impulse Test.

As per Table 2, 82.5% of the participants indicated that they would use case history to identify fall risk factors. However, only 14% of the participants expressed a sense of sufficient knowledge regarding the factors that contribute to the risk of falling among older adults. Therefore, it is uncertain as to what factors participants were considering to be risk factors within the collected case history. Moreover, 50% of the participants would use observation of the patient’s gait and steadiness and questions about history of falls, fear of falling and presence of dizziness or imbalance in their FRS protocol.

Majority of the participants (84%) agreed on the importance of multidisciplinary collaboration for FRS. Physiotherapists (73%) were the most common professionals for referral, followed by ENT (Ear, Nose, Throat) specialists (45%) and General Physicians (GPs) (44%).

### Perceived barriers

In all, 80% of the participants identified the need for additional training as a barrier to implementing FRS. Time constraints and workload were reported by 48%, while 38% highlighted challenges in multidisciplinary collaboration. Further, 38% of the participants indicated discomfort with balance or vestibular testing as a barrier to conducting FRS.

## Discussion

This study explored South African audiologists’ perceived knowledge, attitudes and practices related to FRS. Key findings indicated that while participants reported low levels of perceived knowledge in FRS, they acknowledged its importance within audiology and recognised the critical role audiologists play in fall prevention among older adults.

The inadequate knowledge and training identified in the current study mirrors findings from Greene et al.^22^ and Patterson and Honaker.^19^ Despite some exposure to FRS training, participants still felt underprepared, likely because of the relatively recent inclusion of vestibular training in curricula^35^ and outdated HPCSA guidelines during the study period. There is a need for standardised FRS guidelines and more formal training resources.^36,37^ Aligning with WHO’s global initiative,^38^ developing evidence-based FRS protocols and related training for audiologists should be prioritised. The inclusion of FRS as a routine practice by audiologists necessitates robust training programmes supported by global success stories and empirical evidence. Various initiatives and studies underscore the significance of such training in improving patient outcomes.

The WHO’s guidelines on fall prevention emphasise the profound impact of structured fall risk management programmes. Montero-Odasso et al.^21^ documented global successes, demonstrating reduced fall incidences and improved quality of life for older adults when preventive measures are integrated into healthcare systems. Specific examples of fall risk training have shown tangible benefits. For instance, Criter and Honaker^39^ illustrated that audiologists trained to administer tools such as the Tinetti Assessment Tool and Timed Up and Go (TUG) test significantly enhanced their ability to identify at risk individuals. These interventions facilitated timely referrals to appropriate healthcare providers, directly contributing to reduced fall rates. In Nigeria, Kalu et al.^25^ reported that targeted training programmes for physiotherapists markedly improved their knowledge of fall risk factors and their implementation in clinical practice. This training led to notable reductions in fall incidents among older patients in both community and hospital settings.

Training audiologists in vestibular assessments and specific FRS tools has demonstrated measurable benefits. For example, Greene et al.^22^ and Bassett and Honaker^38^ observed that trained audiologists’ early identification of at-risk patients resulted in timely multidisciplinary interventions. In pilot studies, this approach was associated with a reduction in fall incidents by up to 30%.

Incorporating these insights reinforces the necessity of integrating FRS training into audiology curricula and continued professional development (CPD) programmes. The demonstrated effectiveness of such training highlights the potential for significant contributions by audiologists to fall prevention efforts, ultimately improving patient safety and quality of life. The current study findings highlight several critical implications for audiology practice and the need for FRS training. The low percentage of audiologists with prior knowledge of FRS (11%) and the limited training opportunities during undergraduate studies (<8%) underscore significant gaps in current educational curricula. The interest expressed by participants in further training (98%) suggests an opportunity for targeted professional development initiatives.

Comparisons with international studies reveal that audiologists in SA face similar challenges to their global counterparts. For instance, studies in the United States and Europe, such as those by Bassett and Honaker,^38^ have emphasised the importance of incorporating FRS into routine audiological practice to enhance patient outcomes. Similarly, Criter and Honaker^37^ found that many audiologists lacked confidence in implementing FRS because of insufficient training.

A systematic review by Van Rie et al.^26^ examined professional guidelines and reported practices of audiologists performing FRS with older adults. The review found that many audiologists are not conducting fall risk assessments clinically, primarily because of limited guidance within audiology documentation and inadequate training and knowledge of fall risk factors and measures. Furthermore, a survey conducted by the Canadian Academy of Audiology in 2017^40^ revealed that over 75% of the respondents did not conduct any fall prevention screening tools, despite it being within the scope of practice. This survey suggested that although audiologists support the assessment of fall risk, further education is warranted.

The level of multidisciplinary collaboration reported in SA (84%) is encouraging and provides a strong foundation for improving fall prevention strategies. However, the disparity between participants’ recognition of the importance of FRS (68%) and their comfort in conducting FRS (21%) highlights a need for practical, hands-on training. These findings, consistent with the aforementioned studies, underscore the importance of targeted educational initiatives and professional development opportunities.

The findings also reveal barriers such as time constraints (48%) and challenges with collaboration (38%), which align with Bethlehem^31^ and Wright,^28^ who reported similar constraints in healthcare settings. Addressing these barriers through streamlined processes and clear guidelines could facilitate broader adoption of FRS in audiological practice.

### Strengths and limitations

This study has several limitations that should be acknowledged. The sample size, while representing approximately 7% of registered audiologists in South Africa, may limit the generalisability of the findings. In addition, the self-reported nature of the data introduces the possibility of response bias, as participants may have provided socially desirable answers rather than reflecting their actual practices or knowledge. Selection bias could also have occurred, as audiologists who chose to participate may have had a greater interest in FRS compared to non-respondents. Lastly, the survey instrument, although carefully designed, relied on participant interpretation and self-assessment, which may have introduced information bias. These limitations highlight the need for further research with larger and more diverse samples to validate and expand upon the findings of this study.

### Implications or recommendations

This study highlights several clinical implications for audiology practice and education. The inclusion of FRS in UG curricula is essential to prepare future audiologists to address the needs of older adults effectively. Although FRS has been recently recognised by HPCSA to fall within the scope of practice for audiologists, the current guidelines should be updated to formally incorporate FRS as a standard responsibility that will align with global best practices and ensure ethical care. In addition, the incorporation of standardised FRS tools into existing clinical guidelines and case history forms will streamline implementation and facilitate consistent practice. These recommendations aim to bridge the gap between audiologists’ current practices and the emerging need for comprehensive fall prevention strategies.

Future research should explore the efficacy of targeted training programmes in improving audiologists’ knowledge and practices regarding FRS. Longitudinal studies could evaluate the impact of integrating FRS into routine audiological practice on patient outcomes. In addition, qualitative studies could provide in-depth insights into barriers and facilitators of FRS from both audiologists’ and patients’ perspectives. Finally, comparative studies between SA and other regions could help identify best practices and culturally relevant strategies for fall prevention in diverse healthcare contexts.

## Conclusion

This study underscores the critical role of FRS in audiology practice and highlights the necessity of integrating comprehensive, hands-on FRS training into audiology curricula and professional development programmes. By addressing knowledge gaps and barriers to implementation, the findings advocate for a proactive approach to fall prevention, which can significantly enhance patient safety and quality of life. The incorporation of FRS into routine audiology practice not only aligns with global healthcare priorities but also positions audiologists as pivotal contributors to multidisciplinary fall prevention efforts. In addition, clear objectives have demonstrated the importance of evaluating perceived knowledge and practices while identifying opportunities to advance training. These elements collectively inform evidence-based guidelines and strategies, ensuring audiologists are better equipped to manage fall risks and improve older adults’ quality of life.
